# Pharmacogenomics of Clozapine-induced agranulocytosis: a systematic review and meta-analysis

**DOI:** 10.1038/s41397-022-00281-9

**Published:** 2022-06-16

**Authors:** Farhana Islam, Daniel Hain, David Lewis, Rebecca Law, Lisa C. Brown, Julie-Anne Tanner, Daniel J. Müller

**Affiliations:** 1 Campbell Family Mental Health Research Institute, Centre for Addiction and Mental Health, Toronto, ON Canada; 2 Department of Pharmacology & Toxicology, University of Toronto, Toronto, ON Canada; 3 Myriad Genetics, Salt Lake City, UT USA; 4 Department of Psychiatry, University of Toronto, Toronto, ON Canada

**Keywords:** Predictive markers, Genetic markers

## Abstract

Although clozapine is the most effective pharmacotherapy for treatment-resistant schizophrenia, it is under-utilized, and initiation is often delayed. One reason is the occurrence of a potentially fatal adverse reaction, clozapine-induced agranulocytosis (CIA). Identifying genetic variations contributing to CIA would help predict patient risk of developing CIA and personalize treatment. Here, we (1) review existing pharmacogenomic studies of CIA, and (2) conduct meta-analyses to identify targets for clinical implementation. A systematic literature search identified studies that included individuals receiving clozapine who developed CIA and controls who did not. Results showed that individuals carrying the HLA-DRB1*04:02 allele had nearly sixfold (95% CI 2.20–15.80, p_corrected_ = 0.03) higher odds of CIA with a negative predictive value of 99.3%. Previously unreplicated alleles, *TNFb5*, HLA-B*59:01, *TNFb4*, and *TNFd3* showed significant associations with CIA after multiple-testing corrections. Our findings suggest that a predictive HLA-DRB1*04:02-based pharmacogenomic test may be promising for clinical implementation but requires further investigation.

## Introduction

Schizophrenia is a debilitating condition that affects as many as 20 million people worldwide [1]. Approximately 20–30% of these individuals experience treatment-resistant schizophrenia (TRS), which is characterized by ongoing psychotic symptoms and functional impairments despite adequate trials with different antipsychotic medications [2]. At present, clozapine remains the standard treatment of choice for TRS recommended by international guidelines due to its superior efficacy compared to other existing antipsychotics [3–7]. Despite the abundance of robust evidence supporting the effectiveness of clozapine in improving outcomes for TRS patients, clozapine is underutilized due to concerns about tolerability and monitoring [8], and its initiation is commonly delayed for several years in many countries worldwide, including in the USA and Canada [9–11]. Studies have even suggested that the utilization of clozapine earlier in treatment, rather than waiting for multiple drug failures and subsequent severe TRS, results in better response [12–14]. Further, initiation of clozapine has been shown to reduce healthcare costs by decreasing the number of hospitalizations and shifting care from inpatient to outpatient [15].

The reasons for underuse and delay in clozapine initiation could be attributed to several factors, including highly variable and difficult to predict clinical outcomes. For example, roughly 40–70% of patients on clozapine experience persistent symptoms and remain treatment-resistant [16]. Further, side effects in patients taking clozapine vary greatly, ranging from none or mild to life-threatening side effects [16]. Particularly of concern is the development of clozapine-induced agranulocytosis (CIA), which is defined as an absolute neutrophil count (ANC) < 500 cells/mm^3^. CIA is a severe and potentially fatal neutropenia with an overall prevalence of 0.4% (95% CI: 0.3%, 0.6%) and fatality rate of 0.05% (95% CI: 0.03%, 0.09%) [17]. The World Health Organization’s (WHO) Pharmacovigilance global database, VigiBase, containing more than 140,000 clinician reports of clozapine adverse drug reactions (ADRs) classified in over 5,000 ADR categories, showed that the “broad agranulocytosis” category is the third major cause of fatal outcomes after “broad pneumonia” and “sudden death and cardiac arrests” [18]. Although CIA is a rare hematological condition that represents only 2% of reported fatal outcomes within the VigiBase database [18], the U.S. Food and Drug Administration (FDA) along with the majority of global health authorities have mandated that patients taking clozapine receive regular blood draws to monitor neutrophil count. These authorities also require enrollment in the Clozapine Risk Evaluation and Mitigation Strategy (REMS) Program in order to reduce the risk of clozapine-induced neutropenia.

Existing strategies for regular long-term hematological monitoring in patients taking clozapine have been previously criticized for not being cost-effective, especially given that roughly 80% of CIA cases occur within 18 weeks of clozapine initiation, and after one year of clozapine treatment, incidence of CIA decreases to 0.07% or less [19]. One study reported that frequent and long-term monitoring of white blood cell counts increased quality-adjusted survival by less than one day per patient and was more costly compared to no monitoring [20]. The additional physical burden of regular blood monitoring and its related costs further discourages both patients and clinicians from choosing clozapine and may, in part, account for the suboptimal use of clozapine in clinical practice.

To broaden the usage of clozapine and improve outcomes for patients with TRS, researchers have focused on identifying predictive biomarkers for CIA that could be used to identify individuals most at risk. Although the pathophysiology of CIA remains unclear, twin studies have indicated a genetic component contributing to its development [21, 22]. Numerous genetic association studies have been performed to identify genetic factors that increase the susceptibility to CIA. This is because the discovery of reliable genetic markers for CIA could contribute to the development of a predictive pharmacogenomic test to stratify patients based on risk. Patients identified as low risk will be less susceptible to developing CIA and can safely use clozapine with relaxed hematological monitoring, whereas those identified as high risk for developing CIA can undergo close, routine hematological monitoring or be considered for alternate treatments [23]. The development of such a clinical decision-making tool could minimize the risk and incidence of CIA, reduce costs associated with frequent hematological monitoring, and optimize treatment outcomes for TRS patients.

Therefore, the purpose of the current review and meta-analysis is to [1] review existing pharmacogenomic studies for CIA in patients with TRS [2], conduct meta-analyses on alleles reported to be associated with CIA, and [3] discuss the development of a predictive pharmacogenomic test based on alleles significantly associated with CIA for use within clinical practice.

## Methods

### Search strategy

Using PRISMA guidelines, a systematic literature search was performed using PubMed from database inception date to April 2021 [24]. The following Boolean search string was used: (clozapine AND agranulocytosis). Only peer-reviewed articles published in English and on human participants were considered. As such, two PubMed filters, “Species: Humans” and “Languages: English”, were applied.

### Eligibility criteria and study selection

Included studies were those that compared genetic distributions (reported as variant carrier status) among patients who developed CIA (ANC < 500/μL; cases) against patients who were tolerant to clozapine (i.e., did not develop CIA; controls). Not all studies specified the duration of clozapine treatment or dosage in both cases and controls, therefore, there were no restrictions on duration or dose of clozapine treatment. Further, no age restrictions were implemented. Studies with case-control pairings as described above were included. Case studies, conference proceedings, letters to the editor, narratives, meta-analyses, posters, and systematic reviews were not considered for quantitative analysis, but were mentioned in the text if applicable. Studies which did not report genotypes or carrier-status were not considered.

### Data collection process

Data items collected included the ethnicity or ancestry of subjects, the genetic variant(s) studied, the number of cases who carried at least one copy of the genetic variant, the number of cases who carried no copies of the genetic variant, the number of controls who carried at least one copy of the genetic variant, and the number of controls who carried no copies of the genetic variant. All data items were collected as displayed in each article or its supplementary information.

### Statistical analysis

Principal summary measures included odds ratio (OR), 95% confidence interval (CI), z-score, sensitivity, specificity, negative predictive value (NPV), positive predictive value (PPV), number needed to genotype, and *p* value. NPV was corrected for the prevalence of CIA (prevalence = 0.91%). Haldane correction was used as necessary for the calculation of OR summary measures [25]. Meta-analyses were performed using Review Manager 5.3 (RevMan 5.3, The Cochrane Collaboration), using dichotomous Mantel-Haenszel OR measures with random effects. A random effects model was used under the assumption that the various ancestral populations in the included studies would introduce heterogeneity due to varying patterns of linkage disequilibrium (LD).

LD analysis was performed using haplotype and allele frequency data extracted from http://www.allelefrequencies.net. The calculation of LD statistics was performed as described by Slatkin [26]. Using a bone marrow registry for a Polish population (http://www.allelefrequencies.net/pop6001c.asp?pop_id=3670), LD statistics were calculated for HLA-DRB1*04:02 and HLA-DQB1*05:02.

Bias was not assessed in individual studies; however, findings were corrected for multiple testing using Bonferroni correction and meta-analyses were assessed for heterogeneity using I^2^.

## Results

**Fig. 1:**
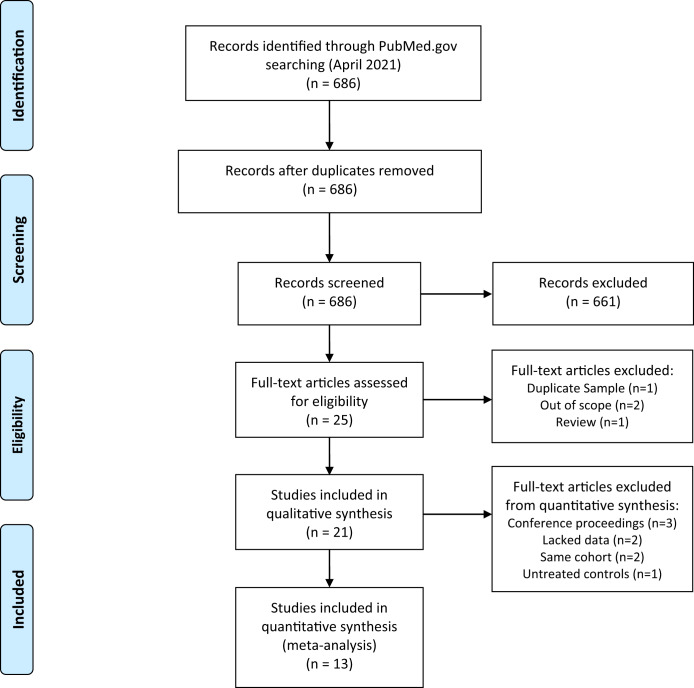
PRISMA diagram (Moher et al., 2009). Preferred Reporting Items for Systematic Reviews and Meta-Analyses (PRISMA) flowchart for identifying clinical studies included in this meta-analysis.

A total of 686 studies were identified, with 661 excluded following the screening of titles and abstracts. After screening and removal of duplicates, 21 studies met the selection criteria for being included into the literature review, and 13 of the 21 studies qualified for inclusion into the meta-analysis. The PRISMA flowchart with details of the search yield is shown in Fig. 1.

The characteristics of the included studies in the meta-analysis are summarized in Table S1. All of the studies included individuals receiving clozapine treatment who demonstrated CIA defined as an ANC < 500/mm^3^ (i.e., <0.5 × 10^9^/L or <500/μL), and comparison or control participants who had not developed any hematotoxic reactions to clozapine. The mean daily dosage of clozapine was 417.0 ± 144.8 mg/d and 482.4 ± 159.0 mg/d for the CIA and comparison groups, respectively, for studies that reported these data.

Of the 13 studies included in the meta-analysis, two were genome-wide association studies (GWAS) [27, 28] and the rest were candidate gene studies conducted in different populations, including Ashkenazi Jewish [29–31], Europeans [27, 32–38], Japanese [28], and others [39]. Of the 13 studies, eight (61.5%) included only non-Jewish European, three (23.1%) included only Jewish, one (7.7%) included a mix of non-Jewish and Jewish Europeans, and one (7.7%) included Japanese samples. One study included participants with diagnoses other than schizophrenia or schizoaffective disorder [29].

Tables 1 and 2 summarize the findings for fifty-three alleles and seven haplotypes, respectively, of individual studies for which no previous replication was found (i.e., these studies reported on allelic markers which have not been investigated in other independent studies). Twelve additional alleles and one additional haplotype were evaluated in at least two studies and were analyzed via meta-analysis shown in Table 3. Therefore, Bonferroni correction for multiple testing (m = 73) was applied to each of the 73-total analyses. After correction for multiple testing, four of the non-replicated alleles remained significant predictors of CIA, including *TNFb5* (OR = 0.08; 95% CI 0.04, 0.20; *p*_c_ = 1.64 × 10^−6^), HLA-B*59:01 (OR = 7.21; 95% CI 3.56, 14.61; *p*_c_ = 3.06 × 10^−6^), *TNFb4* (OR = 7.69; 95% CI 3.55, 16.65; *p*_c_ = 1.71 × 10^−5^), and *TNFd3* (OR = 4.61; 95% CI 2.17, 9.82; *p*_c_ = 5.23 × 10^−3^). None of the non-replicated haplotypes were significant predictors of CIA after Bonferroni correction.

**Table 1:** Summary statistics of individual studies for non-replicated alleles.

Author	Ethnicity	Allele	CIA +	CIA−	Control +	Control−	Sensitivity	Specificity	NPV^a^	PPV^a^	NNG	OR [95% CI]	| Z |	*p* value	p^b,a^
Turbay 1997^c^	European	TNFb5	9	57	43	23	14%	35%	97.8%	0.2%	−2	0.08 [0.04, 0.20]	5.6	2.25E–08	1.64E–06
Saito 2016	Japanese	HLA-B*59:01	19	53	18	362	26%	95%	99.3%	4.9%	3	7.21 [3.56, 14.61]	5.5	4.19E–08	3.06E–06
Turbay 1997^c^	European	TNFb4	48	18	17	49	73%	74%	99.7%	2.5%	3	7.69 [3.55, 16.65]	5.2	2.34E–07	1.71E–05
Turbay 1997^c^	European	TNFd3	51	15	28	38	77%	58%	99.6%	1.6%	3	4.61 [2.17, 9.82]	4.0	7.16E–05	5.23E–03
Lahdelma 2001	Caucasian	HLA-A1	3	23	11	8	12%	42%	98.1%	0.2%	−2	0.09 [0.02, 0.43]	3.1	2.22E–03	0.16
Yunis 1995	Non-Jewish	HLA-DQA1*01:02	15	27	3	29	36%	91%	99.4%	3.4%	3	5.37 [1.40, 20.63]	2.4	0.01	1
Yunis 1995	Ashkenazi Jewish	HLA-DR4	9	1	12	20	90%	63%	99.9%	2.2%	3	15.00 [1.68, 133.56]	2.4	0.02	1
Turbay 1997^c^	Ashkenazi Jewish	HLA-DQA1*03:01	12	12	13	41	50%	76%	99.4%	1.9%	4	3.15 [1.14, 8.70]	2.2	0.03	1
Yunis 1995	Non-Jewish	HLA-DR2	13	8	5	14	62%	74%	99.5%	2.1%	3	4.55 [1.18, 17.52]	2.2	0.03	1
Turbay 1997^c^	Non-Jewish	HLA-DRB1*02	14	26	4	28	35%	88%	99.3%	2.5%	4	3.77 [1.10, 12.93]	2.1	0.03	1
Ostrousky 2003	Jewish	NQO2 372 T > C	14	4	40	40	78%	50%	99.6%	1.4%	6	3.50 [1.06, 11.56]	2.1	0.04	1
Ostrousky 2003	Jewish	NQO2 202 G > A	17	1	54	26	94%	33%	99.8%	1.3%	5	8.19 [1.03, 64.89]	2.0	0.05	1
van der Weide 2017	Dutch	ABCB1 2677 G > T	16	15	167	73	52%	30%	98.6%	0.7%	−13	0.47 [0.22, 0.99]	2.0	0.05	1
Lahdelma 2001	Caucasian	HLA-A28	8	18	1	18	31%	95%	99.3%	5.1%	3	8.00 [0.91, 70.71]	1.9	0.06	1
Dettling 2001	German	HLA-DQB1*02:01	13	17	20	57	43%	74%	99.3%	1.5%	7	2.18 [0.90, 5.27]	1.7	0.08	1
van der Weide 2017	Dutch	ABCB1 3435 C > T	26	5	166	75	84%	31%	99.5%	1.1%	14	2.35 [0.87, 6.36]	1.7	0.09	1
Lahdelma 2001	Caucasian	HLA-A9	6	20	1	18	23%	95%	99.3%	3.9%	4	5.40 [0.59, 49.26]	1.5	0.13	1
van der Weide 2017	Dutch	TNFa -308 G > A	12	19	63	178	39%	74%	99.2%	1.3%	16	1.78 [0.82, 3.88]	1.5	0.14	1
van der Weide 2017	Dutch	Hsp70-2 1267 G > A	22	9	195	43	71%	18%	98.5%	0.8%	−14	0.54 [0.23, 1.25]	1.4	0.15	1
Lahdelma 2001	Caucasian	HLA-B16	6	16	2	17	27%	89%	99.3%	2.3%	4	3.19 [0.56, 18.16]	1.3	0.19	1
Lahdelma 2001	Caucasian	HLA-A11	5	21	1	18	19%	95%	99.2%	3.2%	4	4.29 [0.46, 40.16]	1.3	0.20	1
Lahdelma 2001	Caucasian	HLA-B27	1	21	3	16	5%	84%	99.0%	0.3%	−4	0.25 [0.02, 2.68]	1.1	0.25	1
Yunis 1995	Ashkenazi Jewish	HLA-DQ3	9	1	23	9	90%	28%	99.7%	1.1%	6	3.52 [0.39, 31.95]	1.1	0.26	1
Mosyagin 2005	Caucasian	FcγRIIa R/H	41	7	59	16	85%	21%	99.4%	1.0%	10	1.59 [0.60, 4.20]	0.9	0.35	1
Yunis 1995	Non-Jewish	HLA-DQ1	13	8	14	5	62%	26%	98.7%	0.8%	−8	0.58 [0.15, 2.24]	0.8	0.43	1
Mosyagin 2005	Caucasian	FcγRIIIb NA2/NA1	25	23	44	31	52%	41%	98.9%	0.8%	−16	0.77 [0.37, 1.59]	0.7	0.47	1
Lahdelma 2001	Caucasian	HLA-B18	1	21	2	17	5%	89%	99.0%	0.4%	−5	0.40 [0.03, 4.85]	0.7	0.48	1
Lahdelma 2001	Caucasian	HLA-B37	1	21	2	17	5%	89%	99.0%	0.4%	−5	0.40 [0.03, 4.85]	0.7	0.48	1
Lahdelma 2001	Caucasian	HLA-B12	4	18	2	17	18%	89%	99.2%	1.6%	7	1.89 [0.31, 11.68]	0.7	0.49	1
Lahdelma 2001	Caucasian	HLA-B8	5	17	6	13	23%	68%	99.0%	0.7%	−9	0.64 [0.16, 2.56]	0.6	0.52	1
Lahdelma 2001	Caucasian	HLA-A2	10	16	9	10	38%	53%	98.9%	0.7%	−12	0.69 [0.21, 2.30]	0.6	0.55	1
Lahdelma 2001	Caucasian	HLA-B5	5	17	3	16	23%	84%	99.2%	1.3%	10	1.57 [0.32, 7.66]	0.6	0.58	1
Ostrousky 2003	Jewish	NQO2 -394 G > C	16	1	63	2	94%	3%	98.3%	0.9%	−8	0.51 [0.04, 5.96]	0.5	0.59	1
Lahdelma 2001	Caucasian	HLA-A3	9	17	8	11	35%	58%	99.0%	0.7%	−13	0.73 [0.22, 2.46]	0.5	0.61	1
Lahdelma 2001	Caucasian	HLA-B13	2	20	1	18	9%	95%	99.1%	1.6%	8	1.80 [0.15, 21.57]	0.5	0.64	1
Lahdelma 2001	Caucasian	HLA-B22	2	20	1	18	9%	95%	99.1%	1.6%	8	1.80 [0.15, 21.57]	0.5	0.64	1
van der Weide 2017	Dutch	GSTM1null	16	15	113	125	52%	53%	99.2%	1.0%	60	1.18 [0.56, 2.50]	0.4	0.67	1
Mosyagin 2005	Caucasian	FcγRIIIa F/V	28	20	41	34	58%	45%	99.2%	1.0%	29	1.16 [0.56, 2.41]	0.4	0.69	1
Lahdelma 2001	Caucasian	HLA-A10	2	24	1	18	8%	95%	99.1%	1.3%	11	1.50 [0.13, 17.86]	0.3	0.75	1
Dettling 2001	German	HLA-DQB1*03	16	14	43	34	53%	44%	99.0%	0.9%	−49	0.90 [0.39, 2.11]	0.2	0.81	1
van der Weide 2017	Dutch	GSTT1null	27	4	204	34	87%	14%	99.2%	0.9%	87	1.13 [0.37, 3.42]	0.2	0.84	1
Lahdelma 2001	Caucasian	HLA-B15	4	18	3	16	18%	84%	99.1%	1.0%	24	1.19 [0.23, 6.12]	0.2	0.84	1
Mosyagin 2004	Caucasian	CYPBA C242T	42	38	39	37	53%	49%	99.1%	0.9%	85	1.05 [0.56, 1.97]	0.1	0.88	1
Ostrousky 2003	Jewish	NQO2 -367 A > G	15	2	65	8	88%	11%	99.0%	0.9%	−80	0.92 [0.18, 4.80]	0.1	0.92	1
van der Weide 2017	Dutch	GSTP1 313 A > G	28	3	211	24	90%	10%	99.1%	0.9%	166	1.06 [0.30, 3.76]	0.1	0.93	1
Lahdelma 2001	Caucasian	HLA-A19	4	22	3	16	15%	84%	99.1%	0.9%	−133	0.97 [0.19, 4.95]	0.0	0.97	1
van der Weide 2017	Dutch	GSTA1 -69 C > T	26	5	199	39	84%	16%	99.1%	0.9%	522	1.02 [0.37, 2.82]	0.0	0.97	1
Ostrousky 2003	Jewish	NQO2 1536 C > T	18	0	41	39	100%	49%	100.0%	1.8%	4	35.22 [2.05, 604.37]^d^	2.5^d^	0.01^d^	1^d^
Yunis 1995	Ashkenazi Jewish	HLA-DQB1*03:01	0	16	18	36	0%	67%	98.6%	0.0%	−4	0.06 [0.00, 1.05]^d^	1.9^d^	0.05^d^	1^d^
Lahdelma 2001	Caucasian	HLA-B40	5	17	0	19	23%	100%	99.3%	100.0%	2	12.26 [0.63, 238.00]^d^	1.7^d^	0.10^d^	1^d^
Yunis 1995	Ashkenazi Jewish	HLA-DRB1*11	0	16	13	41	0%	76%	98.8%	0.0%	−4	0.09 [0.01, 1.66]^d^	1.6^d^	0.11^d^	1^d^
Lahdelma 2001	Caucasian	HLA-B17	0	22	1	18	0%	95%	99.0%	0.0%	−2	0.27 [0.01, 7.13]^d^	0.8^d^	0.44^d^	1^d^
van der Weide 2017	Dutch	NQO1 609 C > T	31	0	234	7	100%	3%	100.0%	0.9%	9	2.01 [0.11, 36.14]^d^	0.5^d^	0.63^d^	1^d^

*CIA* clozapine-induced agranulocytosis, *CIA+*, number of variant positive CIA subjects, *CIA−* number of variant negative CIA subjects, *Control+* number of variant positive control subjects, *Control−* number of variant negative control subjects, *NNG* number needed to genotype, *NPV* negative predictive value, *OR* odds ratio, *PPV* positive predictive value.

^a^NPV and PPV were corrected for the prevalence of CIA in the US.

^b^Bonferroni correction (*m* = 73) was applied based on the number of alleles/haplotypes analyzed in this review.

^c^Study included both Jewish and Non-Jewish individuals.

^d^Haldane correction was applied for case-control pairings which had 0 subjects in at least one cell.

**Table 2:** Summary statistics of individual studies for non-replicated haplotypes.

Author	Ethnicity	Haplotype	CIA +	CIA−	Control +	Control−	Sensitivity	Specificity	NPV^a^	PPV^a^	NNG	OR [95% CI]	| Z |	*p* value	p^b,a^
Yunis 1995	Ashkenazi Jewish	HLA-B38, -DR4, -DQ3	9	1	6	26	90%	81%	99.9%	4.2%	2	39.00 [4.12, 369.53]	3.2	0.00	0.10
Yunis 1995	Ashkenazi Jewish	DRB1*04:02, DRB4*01:01, DQB1*03:02, DQA1*03:01	7	9	6	48	44%	89%	99.4%	3.5%	3	6.22 [1.69, 22.88]	2.8	0.01	0.43
Turbay 1997	European	HLA-DRB1*0402, DRB4*0101, DQB1*0302, DQA1*0301, HSP70-2*A, HSP70-1*9, TNFe3, TNFd3, TNFa(0308)*1,TNFbn(A/G)*2, TNFa10, TNFb4, HLA-B38	12	52	4	62	19%	94%	99.2%	2.8%	4	3.58 [1.09, 11.76]	2.1	0.04	1
Theodoropoulou 1997	Non-Jewish	HLA-B16, -DR4, -DQ3	1	2	1	39	33%	98%	99.4%	10.9%	3	19.50 [0.87, 439.35]	1.9	0.06	1
Turbay 1997	European	HLA-DRB1*02, DRB5*02, DQB1*0502, DQA1*0102, HSP70-2*A, HSP70-1*9, TNFe3, TNFd3, TNFa(0308)*1, TNFbn(A/G)*2, TNFa11, TNFb4	10	54	0	66	16%	100%	99.2%	100.0%	2	25.62 [1.47, 447.25]^c^	2.2^c^	0.03^c^	1^c^
Yunis 1995	Non-Jewish	HLA-DRB1*16:01, -DRB5*02, -DQB1*05:02, -DQA1*01:02	10	32	0	32	24%	100%	99.3%	100.0%	2	21.00 [1.18, 373.52]^c^	2.1^c^	0.04^c^	1^c^
Yunis 1995	Non-Jewish	HLA-B7, -DR2, -DQ1	5	16	0	19	24%	100%	99.3%	100.0%	2	13.00 [0.67, 252.99]^c^	1.7^c^	0.09^c^	1^c^

*CIA* clozapine-induced agranulocytosis, *CIA+* number of variant positive CIA subjects, *CIA−* number of variant negative CIA subjects, *Control* *+*  number of variant positive control subjects, *Control−* number of variant negative control subjects, *NNG* number needed to genotype, *NPV* negative predictive value, *OR* odds ratio, *PPV* positive predictive value.

^a^NPV and PPV were corrected for the prevalence of CIA in the US.

^b^Bonferroni correction (*m* = 73) was applied based on the number of alleles/haplotypes analyzed in this review.

^c^Haldane correction was applied for case-control pairings which had 0 subjects in at least one cell.

**Table 3:** Summary statistics of meta-analyses.

Authors	Allele/Haplotype	CIA +	CIA−	Control +	Control−	Sensitivity	Specificity	NPV^a^	PPV^a^	NNG	OR [95% CI]	| Z |	I^2^	*p* value	p^b,a^
Dettling 2001, Turbay 1997	HLA-DRB1*04:02	14	40	8	123	26%	94%	99.3%	3.8%	3	5.89 [2.20, 15.80]	3.5	0%	4.00E–04	0.03
Legge 2016, Yunis 1995, van der Weide 2017, Athanasiou 2011	HLA-DQB1*05:02	33	178	9	521	16%	98%	99.2%	7.8%	2	7.12 [1.91, 26.51]	2.9	53%	3.00E–03	0.22
Theodoropoulou 1997, Yunis 1995	HLA-DR2, -DQ1	15	9	18	41	63%	69%	99.5%	1.8%	4	5.40 [1.58, 18.43]	2.7	0%	0.01	0.511
Dettling 2001, Yunis 1995	HLA-DRB5*02	13	59	2	107	18%	98%	99.2%	8.3%	2	6.44 [1.57, 26.39]	2.6	0%	0.01	0.73
Dettling 2001, Yunis 1995	HLA-DRB1*16:01	13	59	5	104	18%	95%	99.2%	3.5%	3	3.62 [1.15, 11.45]	2.2	0%	0.03	1
Yunis 1995, Valevski 1998, Dettling 2001	HLA-B38	27	29	22	137	48%	86%	99.5%	3.1%	3	10.01 [1.13, 88.55]	2.1	82%	0.04	1
Ostrousky 2003, van der Weide 2017	NQO2 1541 G > A	40	9	154	167	82%	52%	99.7%	1.5%	7	7.16 [0.52, 98.34]	1.5	70%	0.14	1
Dettling 2001, Turbay 1997	HLA-DQB1*03:02	16	38	20	111	30%	85%	99.2%	1.8%	6	2.31 [0.53, 10.09]	1.1	71%	0.26	1
Mosyagin 2004, van der Weide 2017	CYBA 640 A > G	78	31	246	70	72%	22%	98.8%	0.8%	−16	0.70 [0.36, 1.38]	1.0	27%	0.31	1
Lahdelma 2001, Yunis 1995	HLA-B7	10	32	5	33	24%	87%	99.2%	1.6%	6	2.17 [0.14, 34.50]	0.6	75%	0.58	1
Dettling 2001, Yunis 1995	HLA-DRB4	20	26	53	78	43%	60%	99.1%	1.0%	42	1.27 [0.35, 4.55]	0.4	69%	0.72	1
Dettling 2001, Lahdelma 2001	HLA-B35	10	42	19	87	19%	82%	99.1%	1.0%	52	1.08 [0.45, 2.57]	0.2	0%	0.87	1
Mosyagin 2004, van der Weide 2017	MPO −463 G > A	42	70	125	193	38%	61%	99.1%	0.9%	−69	1.03 [0.63, 1.68]	0.1	0%	0.92	1

*CIA* clozapine-induced agranulocytosis, *CIA+* number of variant positive CIA subjects, *CIA−* number of variant negative CIA subjects, *Control+* number of variant positive control subjects, *Control−* number of variant negative control subjects, *NNG* number needed to genotype, *NPV* negative predictive value, *OR* odds ratio, *PPV* positive predictive value.

^a^NPV and PPV were corrected for the prevalence of CIA in the US.

^b^Bonferroni correction (*m* = 73) was applied based on the number of alleles/haplotypes analyzed in this review.

After correction for multiple testing (m = 73), one of the meta-analyzed alleles (Table 3) remained a significant predictor of CIA, HLA-DRB1*04:02 (OR = 5.89; 95% CI 2.20, 15.80; *p*_c_ = 0.03). The sensitivity and the specificity values of the HLA-DRB1*04:02 allele for prediction of CIA were 26.0% and 94.0%, respectively. The PPV and NPV were estimated to be 3.8% and 99.3%, respectively. The number of new clozapine users needed to genotype to prevent one case of CIA is three in individuals of European ancestry, which may vary in other ancestral groups. Forest plots for each meta-analysis are available in the supplementary materials (Fig. S1).

Additional alleles have reached genome wide significance such as rs149104283 [27, 40], rs3129891 [40], rs41549217 [40], and *HLA-B* 158 T [41]. However, *HLA-B* 158 T was not found to be significantly associated with CIA in a second study [42].

## Discussion

We systematically summarized and quantified available evidence on genetic variants contributing to CIA and conducted several meta-analyses. We found that one genetic variant within the human leukocyte antigen (*HLA*) locus (major histocompatibility complex [MHC] in humans) was significantly associated with CIA after correction for multiple testing. Specifically, individuals carrying the HLA-DRB1*04:02 allele had nearly sixfold (95% CI 2.20, 15.80) increased odds of CIA. For this variant, the probability that CIA was not present in individuals without the HLA-DRB1*04:02 allele (i.e., NPV) was 99.3%, corrected for the prevalence of CIA in the USA. A high NPV indicates potential clinical utility of the HLA-DRB1*04:02 allele in stratifying patients based on risk of developing CIA, with those that are low risk (i.e., non-carriers of the variant) being suitable candidates for clozapine treatment with a relaxed hematological monitoring schedule and those that are not low risk (i.e., carriers of the variant) monitored more closely while on clozapine or considered for alternative treatment options.

HLA-DRB1*04:02 genotyping prior to the initiation of clozapine, if clinically implemented, would not be the first HLA predictive test for assessing risk of drug-related adverse reactions. Currently, the U.S. FDA recommends prospective screening for specific HLA alleles that are strongly associated with hypersensitivity reactions to carbamazepine, abacavir, and allopurinol prior to their initiation in populations where the allele is common [43–45]. In comparison to these existing predictive tests, the NPV of HLA-DRB1*04:02 (99.3%) is higher than the NPV of HLA-B*15:11 and HLA-B*57:01 genotyping for carbamazepine (98.9%) [46] and abacavir (82%) [47] hypersensitivity reactions, respectively, and about the same as the HLA-B*58:01 test (99.0%) for allopurinol-induced severe cutaneous adverse reactions (SCARs) [48]. Although the PPV of HLA-DRB1*04:02 was low (3.8%), indicating its weak ability to identify individuals who did indeed have CIA and avoiding false negatives, it is higher than the PPV of HLA-B*15:11 (1.0%) genotyping for carbamazepine-induced SCARs [49].

Given that CIA is potentially fatal, the low PPV of HLA-DRB1*04:02 genotyping is greatly outweighed by the very high NPV. Furthermore, the sensitivity of HLA-DRB1*04:02 for the prediction of CIA is higher than that of the HLA-A*31:01 (23%) [50] and HLA-B*15:11 (14%) [46] alleles for the prediction of carbamazepine-induced SCARs, but lower than of the HLA-B*58:01 (100%) [51] and HLA-B*57:01 (51%) [47] alleles for the prediction of allopurinol- and abacavir-induced hypersensitivity, respectively. The specificity of HLA-DRB1*04:02 (94%) was slightly lower than that of HLA-A*31:01 (95%) [50] and HLA-B*15:02 (99%) [46] for carbamazepine and of HLA-B*57:01 (96%) [47] for abacavir hypersensitivity, but higher than that of the HLA-B*58:01 allele (88%) for allopurinol-induced SCARs [51]. Therefore, the predictive value and validity of an HLA-DRB1*04:02 screening test for assessing patient risk of CIA are comparable to existing HLA predictive tests that are currently used clinically.

Although HLA-DRB1*04:02 appears to be a promising biomarker for a predictive pharmacogenomic test for CIA based on our results, it is important to note that the clinical utility of a SNP-based predictive test will differ across populations, since allele frequencies and different patterns of LD in associated regions vary substantially between ancestral groups. Given that the two studies included in the meta-analysis of HLA-DRB1*04:02 were conducted in small non-Jewish German (CIA = 30, Controls = 77) [38] and Ashkenazi Jewish (CIA = 12, Controls = 18) [30] samples, little is known about the predictive value of the HLA-DRB1*04:02 allele in other European and non-European populations. The HLA-DRB1*04 allele of the *HLA-DRB1* gene, encoding the polymorphic beta chain of the HLA-DR antigen-binding cell surface receptor, has been reported to be less frequent in Sub-Saharan Africans (0.022) compared to Europeans (0.177), Native Americans of North America (0.496), Oceanians (0.087), and Southeast Asians (0.129) [52]. HLA-DRB1*04:02 represents the second HLA-DRB1*04 allele within the serologically defined HLA-DR4 antigen family. Data on the allele frequency across populations for HLA-DRB1*04:02 is currently lacking. Furthermore, certain alleles within the HLA region are inherited in a tight cluster as conserved haplotypes, which often varies among different population groups. This means that the HLA-DRB1*04:02 allele may be in LD with other variants that is specific to the study population, and therefore may not show an association with CIA in other ancestral groups. Therefore, the association between the HLA-DRB1*04:02 allele and CIA susceptibility warrants investigation in different ancestral groups in future studies for the application of an HLA-DRB1*04:02-based predictive test for CIA to be relevant across populations.

There were three GWAS on CIA identified by our search. Of the three, two identified GWAS on CIA included the same sample of patients from the Clozapine-Induced Agranulocytosis Consortium (CIAC) (*n* = 161 CIA patients). Given that the statistical analysis assumes independent samples, only the most recent GWAS study of the two was included in the meta-analysis to avoid increased Type 1 error and biased effect estimates. The GWAS by Goldstein et al. (2014) showed a significant association between two MHC loci with CIA, *HLA-DQB1* (126Q) (OR = 0.19, 95% CI 0.12–0.29) and *HLA-B* (158 T) (OR = 3.3, 95% CI 2.3–4.9) [41]. *HLA-DQB1* (126Q) is in strong LD with HLA-DQB1*05:02, the most common HLA high-risk allele for CIA [29, 53], and with *HLA-DQB1* 6672 G > C, also previously reported to be associated with risk for CIA [39]. Results from our meta-analysis showed that HLA-DQB1*05:02 (OR = 7.12, 95% CI 1.91–26.51) was significantly associated with CIA, before Bonferroni corrections were applied (Table 3). Furthermore, Legge et al. (2017) [27] and Konte et al. (2021) [54] provided independent replications for the association between *HLA-DQB1* 6672 G > C and CIA risk in individuals of European ancestry. These results implicate *HLA-DQB1* in the pathophysiology of CIA and indicates that polymorphisms within this gene may be associated with risk of CIA in European populations.

In the GWAS by Legge et al. (2017), an association between genes at chromosome 12p12.2 with CIA was reported in a sample of European patients with the top SNP being rs149104283 (OR = 4.32, *P* = 1.79 × 10^−8^), which is intronic to transcripts of the solute carrier organic anion transporter genes, *SLCO1B3* and *SLCO1B7* [27]. A replication analysis was conducted by Saito et al. (2017) in a Japanese sample as a part of the Clozapine Pharmacogenomics Consortium of Japan, which found no significant association of 12p12.2 with CIA [55]. Instead, in their GWAS, Saito et al. (2016) showed HLA-B*59:01 (OR = 10.7, 95% CI 4.8–22.4) as a risk factor for CIA in a sample of Japanese patients with schizophrenia (CIA = 50, Controls = 2905) [28]. A combined GWAS meta-analysis in a sample of patients of Chinese ancestry identified a nominal association between rs11753309 near *HLA-B* and clozapine-induced neutropenia; however, this GWAS was not included in the meta-analysis as the results are not specific to CIA [56]. Findings from these GWAS taken together demonstrate that risk alleles for CIA may vary by ancestral group given that some variants lie in variation-rich genomic regions and demonstrate large differences in allele frequencies across populations, and population-specific recombination sites contribute to the high diversity of haplotypes further influencing CIA risk [54, 57].

Several well-known alleles and genetic variants are localized within the MHC region and show LD; therefore, it complicates whether conclusions about specific associations between *HLA* alleles with CIA represent a true genetic association or whether the *HLA* allele is in LD, or closely located to the true causative gene [58]. Goldstein et al. (2014) found that HLA-DRB1*04:02 and HLA-DQB1*05:02 were not in strong LD according to r^2^, yet the D’ between these two alleles may be quite high [41]. Limited haplotype frequency data is available for HLA-DRB1*04:02 and HLA-DQB1*05:02, including a bone marrow registry for a Polish population whose LD statistics were calculated. Given a high enough D’, the association between HLA-DRB1*04:02 and CIA may not be sufficiently independent from that of HLA-DQB1*05:02 and makes it difficult to conclude the respective contribution of a given allele to predisposition to CIA. Therefore, further analysis is required to confirm this in other populations, especially considering the rarity of this haplotype in the Polish population.

Previously unreplicated alleles, *TNFb5* (OR = 0.08; 95% CI 0.04–0.20), *TNFb4* (OR = 7.69; 95% CI 3.55–16.65), and *TNFd3* (OR = 4.61; 95% CI 2.17–9.82), showed significant associations with CIA after corrections for multiple testing. *TNF* microsatellites *d3* and *b4* were associated with increased susceptibility to CIA, while microsatellite *b5* showed a protective effect in both Jewish and non-Jewish individuals with schizophrenia [30]. *TNF* are immune-regulating non-*HLA* genes in the MHC region located between the complement cluster region and the *HLA-B* gene, which has more recently been demarcated as the Class IV region, and have shown a LD with *HLA-B* and *HLA-DR* alleles [59]. As a result, for studies showing an association between *TNF* or *HLA-B* alleles with CIA noted above, strong LD between these genes complicates unraveling the relative contributions of genetic variation in the *TNF* locus or the *HLA-B* locus to CIA.

The limitations of the present study include a lack of bias analysis, which may lead to an overestimation of the true effect size of the results, since studies with higher effects are more likely to be published, and thus be included in the meta-analysis. To reduce the effects of publication bias, we performed a comprehensive search to identify all relevant, published literature. An additional limitation is that we cannot confirm with certainty that the sample in the GWAS conducted by Legge et al. (2016) does not overlap with samples in the other studies included in this meta-analysis. For the GWAS by Saito et al. (2016), since it is the only study conducted in patients of Japanese ancestry, it can be safely deduced that it does not overlap with the other studies which are conducted in patients of European ancestry (Table S1). Finally, translating findings from pharmacogenomic investigations, such as the present study, into clinical practice may not necessarily increase the usage of clozapine to treat TRS patients, but may instead be a deterrent to its usage as was the case with carbamazepine, which was substantially less prescribed to patients with epilepsy and bipolar disorder following the introduction of HLA-B*15:02 screening in Taiwan to prevent carbamazepine-related SCARs [60].

## Conclusion

Currently, there is no predictive test for CIA. For a predictive test to be useful within clinical practice, it should reliably be able to identify individuals who are at low risk of developing CIA in non-carriers of the risk allele, such that hematological monitoring is either unnecessary or reduced in frequency. NPV or the proportion of patients with a negative test (i.e. non-carriers of the risk allele) who truly do not have the condition is a reliable diagnostic for the clinical usefulness of a predictive test [61]. Test results with a high NPV are useful to clinicians when considering treatments which can potentially be unnecessary, costly or even risky, such as clozapine pharmacotherapy [61]. Based on the results of the meta-analyses, the HLA-DRB1*04:02 variant demonstrates a potential for pharmacogenomic prediction of CIA within clinical practice with a high NPV (99.3%). Estimates of NPV, PPV, sensitivity, and specificity for HLA-DRB1*04:02 genotyping for CIA risk assessment are comparable to other existing HLA screening tests for drug-induced hypersensitivity reactions that are used clinically. However, since allele frequencies and haplotypes vary substantially by ancestral groups, further research is needed to investigate the association between the HLA-DRB1*04:02 allele and CIA susceptibility in different populations. Furthermore, the results of the meta-analysis indicate immunogenetic variations, specifically relating to the MHC genomic region, may be involved in the pathophysiology of CIA, and therefore are potential targets for identifying other genetic markers for CIA.

Pharmacogenomic investigations to date suggest the involvement of multiple genetic variations with varying levels of impact on CIA. Therefore, further research is necessary to identify reliable and reproducible genetic variants in diverse populations with large effects related to CIA that can be incorporated into a predictive pharmacogenomic test. Clinical application of predictive pharmacogenomic tests with high NPV may increase the safe utilization of clozapine, decrease costs associated with regular long-term hematologic monitoring, and most importantly, improve patient outcomes.

## Supplementary information

Supplementary Materials

## Data Availability

All data generated or analysed during this study are included in this published article and its supplementary information files.
